# Prevalence of Cervical Pap Smear Epithelial Abnormalities in Iraqi Women and Its Correlation with Histopathology

**DOI:** 10.30699/ijp.2025.2064544.3490

**Published:** 2025-11-11

**Authors:** Mais Mohammed Salim, Iftikhar Kudair Abbas, Ebtihal Chiad Abbas, Kaswer Musa Jaafar, Rana Talib Fakher

**Affiliations:** 1 Department of Pathology and Forensic Medicine, Faculty of Medicine, University of Kufa, Najaf Governorate, Iraq; 2 Department of Medical Microbiology, Faculty of Medicine, University of Kufa, Najaf Governorate, Iraq

**Keywords:** Cervical Cancer Screening, Pap Smear, Diagnostic Accuracy

## Abstract

**Background & Objective:**

Cervical carcinoma is the fourth most common malignancy among women worldwide, with a disproportionately high incidence and mortality in developing countries, including Iraq, where 320 new cases and 62 deaths were reported in 2023. Although the Papanicolaou (Pap) smear remains the cornerstone of screening, findings across Arab populations have been inconsistent, underscoring the need for region-specific data. This study aimed to determine the prevalence and patterns of cervical epithelial cell abnormalities and assess the diagnostic accuracy of Pap smears in Najaf, Iraq.

**Methods:**

This retrospective, cross-sectional study was conducted over ten years (December 2014–December 2024) and included 3522 cervical Pap smears from women aged 16 to 80 years (mean, 36.97 ± 10.62 years) obtained at a private medical laboratory. Samples were classified according to the 2014 Bethesda System.

**Results:**

Of 3522 samples, epithelial cell abnormalities were identified in 192 (5.45%). Atypical squamous cells of undetermined significance (ASC-US) were the most frequent abnormality (3.78%). The highest prevalence was observed among women aged 40 to 60 years. A strong cytohistopathologic correlation was noted. The Pap test demonstrated a sensitivity of 76.19%, specificity of 80.30%, and overall diagnostic accuracy of 79.31%.

**Conclusion:**

The 5.45% prevalence of epithelial abnormalities, predominantly ASC-US, highlights the ongoing need for active cervical cancer screening programs. The significant concordance between cytologic and histopathologic findings confirms the diagnostic reliability of the Pap smear. Further studies are warranted to characterize local cytologic patterns and identify prevalent HPV genotypes to inform HPV vaccination and targeted prevention strategies.

## Introduction

Cervical carcinoma constitutes a major global health burden, ranking as the fourth most frequently occurring malignancy among women, with approximately 660,000 new cases and 350,000 deaths reported worldwide in 2022. The highest incidence and mortality rates are observed in countries with low socioeconomic indices, particularly in sub-Saharan Africa, Central America, and Southeast Asia (1). In contrast, incidence rates in Western Asia are substantially lower, showing a 7- to 10-fold reduction (2). In Iraq, epidemiological data from 2023 documented 320 new cases and 62 deaths attributed to cervical carcinoma (3).

Multiple risk factors have been implicated in the etiology of cervical cancer, including tobacco smoking, early onset of sexual activity, multiple lifetime sexual partners, and prolonged oral contraceptive use (4–6). Of particular etiologic significance is infection with human papillomavirus (HPV), which represents the primary causative agent in cervical carcinogenesis (7). The progression from precancerous cellular alterations to invasive malignancy is typically slow, often spanning a decade or more. This extended latency period provides a crucial window for early detection and intervention, significantly improving patient outcomes (8).

Papanicolaou (Pap) smear cytology remains the most reliable and widely used screening method for cervical cancer, serving as the clinical gold standard (9). Its widespread adoption is attributed to its simplicity, noninvasiveness, low morbidity, and cost-effectiveness in detecting benign, premalignant, and malignant cervical lesions (10). The Bethesda System provides a standardized nomenclature for reporting Pap test results and ensures consistent diagnostic interpretation (11). Implementation of organized, population-based Pap smear screening programs has been shown to facilitate earlier diagnosis and reduce both morbidity and mortality associated with cervical cancer.

Despite these advances, approximately 85% of cervical cancer–related deaths occur in developing countries, largely due to the absence of structured screening and HPV vaccination programs, as well as limited public awareness regarding the importance of regular cervical screening (12,13). Although extensive studies have been conducted in Western populations, research from Arab regions has shown variability in the prevalence and patterns of cervical cytological abnormalities and their association with different risk factors (14–16).

The present study was designed to determine the prevalence and distribution patterns of cervical epithelial lesions in Iraqi women and to evaluate the diagnostic accuracy of Pap smear cytology through correlation with histopathologic findings.

## Materials and Methods

This study employed a ten-year retrospective, cross-sectional observational design conducted at a private medical laboratory in Najaf, Iraq, to evaluate cervical cytological specimens collected and processed between December 2014 and December 2024. Owing to its retrospective nature, separate informed consent was not required. The study protocol was reviewed and approved by the Institutional Ethics Committee.

The study population included women aged 16 to 80 years (mean age, 36.97 ± 10.62 years) who presented with various gynecologic symptoms such as vaginal discharge, postcoital bleeding, and intermenstrual bleeding. Cervical specimens were obtained by qualified gynecologists using two distinct collection methods:

1. Conventional method: The elongated portion of an Ayre spatula was inserted into the cervical os and rotated 360° at the squamocolumnar junction. The collected material was gently spread onto a clean glass slide and immediately fixed in 95% ethyl alcohol.
2. Liquid-based cytology method: A cytobrush was used to sample the cervix, and the collection device was then immersed into a BD SurePath™ liquid-based Pap test vial (Becton, Dickinson & Co., Franklin Lakes, NJ, USA).

Cytologic evaluation was performed by a board-certified histopathologist, and findings were classified according to the 2014 Bethesda System. All smears exhibiting cytologic abnormalities were subjected to secondary review for diagnostic confirmation. Patient clinical data were obtained from archived cytopathology reports in the laboratory information system.

Specimens were excluded if they originated from previously diagnosed cases of cervical carcinoma, were inadequate for cytologic evaluation, or were collected from the vaginal vault rather than the cervix.

Statistical analyses were conducted using the Statistical Package for the Social Sciences (SPSS), Version 20 (IBM Corp., Armonk, NY, USA). Continuous variables were presented as means ± standard deviations, whereas categorical variables were expressed as frequencies and percentages. The prevalence of cytologic lesions was reported as a percentage. Associations between cervical lesions and other categorical variables were analyzed using the χ² test or Fisher exact test, as appropriate. A *p* < 0.05 was considered statistically significant for all comparisons.

## Results

 A total of 3522 cervical cytology samples obtained from women aged 16 to 80 years were retrospectively analyzed. The overall cytological findings are summarized in Tables 1 and 2. Of these, 3330 samples (94.55%) demonstrated benign cytological features, whereas 192 samples (5.45%) exhibited epithelial cell abnormalities.

**Fig. 1.**
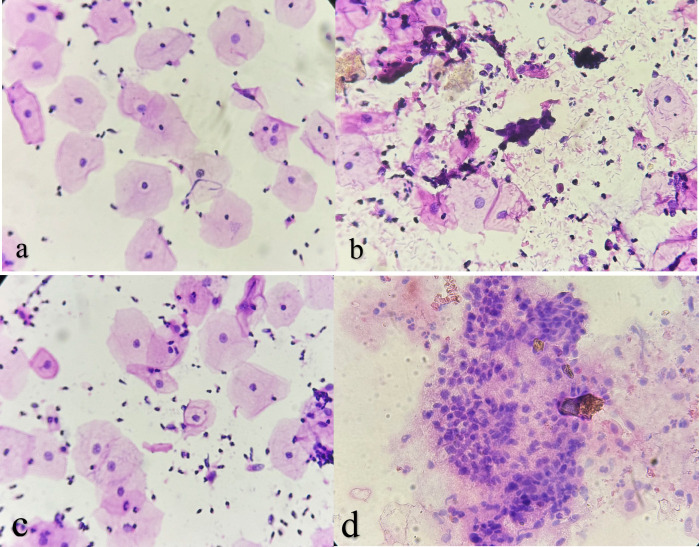
Cervical Pap smears showing: (a) atypical squamous cells of undetermined significance (ASC-US); (b) atypical squamous cells—cannot exclude a high-grade squamous intraepithelial lesion (ASC-H); (c) low-grade squamous intraepithelial lesion (LSIL); (d) high-grade squamous intraepithelial lesion (HSIL).

The most frequent abnormality was Atypical Squamous Cells of Undetermined Significance (ASC-US), detected in 133 cases (3.78%). Other abnormal findings included Atypical Squamous Cells—Cannot Exclude High-Grade Squamous Intraepithelial Lesion (ASC-H) in 24 cases (0.68%), High-Grade Squamous Intraepithelial Lesion (HSIL) in 13 cases (0.37%), Low-Grade Squamous Intraepithelial Lesion (LSIL) in 11 cases (0.31%), and Atypical Glandular Cells—Not Otherwise Specified (AGC-NOS) in 11 cases (0.31%) (Figure 1, Table 3).

Clinically, most patients with abnormal Pap cytology presented with a normal-appearing cervix; the only notable finding was cervical congestion. The highest prevalence of epithelial abnormalities occurred in the 40–60-year age group (Table 4).

Among 59 cases with significant epithelial abnormalities, follow-up histopathologic biopsy results were available for 29 patients. The correlation between cytologic and histopathologic diagnoses in these cases is shown in Table 5. A statistically significant association was observed between Pap smear and histopathologic findings (p = 0.036).

The overall sensitivity, specificity, positive predictive value (PPV), negative predictive value (NPV), and diagnostic accuracy of Pap cytology were 76.19%, 80.30%, 55.17%, 91.38%, and 79.31%, respectively. The diagnostic performance of Pap cytology for individual epithelial lesion categories is detailed in Table 6.

**Table 1:** Age groups distribute the total cases.

Age groups	No. of cases
11-20	90
21-30	972
31-40	1210
41-50	919
51-60	260
61-70	54
71-80	17
Total	3522

**Table 2:** Distribution of normal and abnormal Pap smears by age group.

Age groups	Abnormal PAP	Normal PAP	No. of cases
11-20	3	87	90
21-30	10	962	972
31-40	31	1179	1210
41-50	70	849	919
51-60	55	205	260
61-70	18	36	54
71-80	5	12	17
Total	192	3330	3522

r = 0.338553

**Table 3:** Distribution of cervical epithelial cell abnormalities.

PAP abnormality	No. of cases	%
ASCU-S	133	(3.776%)
ASC-H	24	(0.681%)
LSIL	11	(0.312%)
HSIL	13	(0.369%)
AGC-NOS	11	(0.312%)
Total	192	(5.45%)

**Table 4:** Distribution of abnormal epithelial lesions by age group.

Age group (years)	ASC-US	ASC-H	LSIL	HSIL	AGC-NOS
11–20	3 (1.56%)	0	0	0	0
21–30	9 (4.69%)	1 (0.52%)	0	0	0
31–40	16 (8.33%)	3 (1.56%)	2 (1.04%)	1 (0.52%)	9 (4.69%)
41–50	52 (27.09%)	8 (4.17%)	4 (2.08%)	4 (2.08%)	2 (1.04%)
51–60	39 (20.31%)	7 (3.65%)	3 (1.57%)	6 (3.13%)	0
61–70	11 (5.73%)	4 (2.08%)	2 (1.04%)	1 (0.52%)	0
71–80	3 (1.56%)	1 (0.52%)	0	1 (0.52%)	0
Total	133(69.27%)	24(12.50%)	11(5.73%)	13(6.77%)	11(5.73%)
χ² = 47.085; p = 0.00327					

Chi-square=47.085

*P*-value=0.00327

**Table 5:** Relationship between Pap smear findings and histopathological diagnosis.

PAP smear finding	Cervicitis	CINI	CINII/III	SCC	Adenocarcinoma	Total
LSIL	5	3	2	0	0	10
HSIL	0	1	10	1	0	12
AGC-NOS	3	1	0	0	3	7
Total	8	5	12	1	3	29

P value<0.05

P-value=0.036

**Table 6:** Statistical values of Pap smear for different cervical epithelial lesions in percentage.

Particulars	LISL	HISL	AGC-NOS	Total
Sensitivity	60%	83.333%	75%	76.190%
Specificity	70.833%	88.235%	84%	80.303%
PPV	30%	83.333%	42.857%	55.172%
NPV	89.47%	88.235%	95.454%	91.379%
Diagnostic accuracy	68.965%	86.206%	82.758%	79.310%

*P* value<0.05

**Fig. 2:**
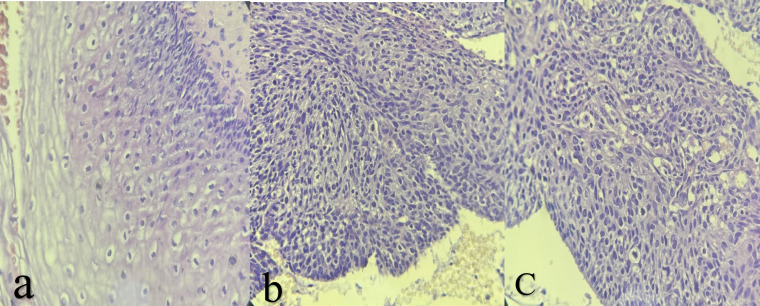
**(a) **Histopathological picture of CIN1 (H and E, X40); **(b)** Histopathological picture of CIN2 (H and E, X40); **(c)** Histopathological picture of CIN3 (H and E, X40).

## Discussion

This study aimed to determine the prevalence and patterns of cervical epithelial lesions among Iraqi women using Pap smear cytology, with results classified according to the 2014 Bethesda System. The overall detection rate of epithelial cell abnormalities was 5.45% (192 of 3522 cases). This prevalence is broadly consistent with findings reported in neighboring Middle Eastern populations, where rates range from 3.8% in Jordan (17), 4.3% in Kuwait (15), 4.89% in the United Arab Emirates (14), 3.7% in Oman (18), 4% in Iran (19), and 4.7–7.9% in various regions of Saudi Arabia (20). These similarities may be related to relatively conservative sexual behaviors and sociocultural norms prevalent in these regions, which could mitigate exposure to certain risk factors.

However, the prevalence in this study exceeds that reported in some European countries, including Turkey (2.8%) (21), Italy (2.4%) (22), and Belgium (3.7%) (23), yet remains lower than that observed in several other countries such as Russia (9.8%) (24), Egypt (7.8%) (25), Romania (5.9%) (26), and India (6.3%) (27). Variability across studies may result from differences in diagnostic criteria, laboratory quality control, sample size, and the influence of regional risk factors such as early sexual debut, multiparity, and HPV prevalence.

In agreement with most published literature, ASC-US was the most common epithelial abnormality, while LSIL was the least frequent (28), consistent with previous findings in Iraq (29). The prevalence of ASC-US (3.77%) in this study was slightly higher than that reported in Kuwait (2.37%) (15), the UAE (2.5%) (14), and Saudi Arabia (2.9%) (20), but lower than that reported in Oman (4.8%) (18). Conversely, the prevalence of LSIL (0.31%) and HSIL (0.37%) was lower than in Kuwait (0.9% and 0.22%) (15), southwestern Saudi Arabia (1.3% and 0.68%) (30), and Oman (1.4% and 0%) (18). The prevalence of AGC-NOS (0.31%) and ASC-H (0.68%) in the present study was slightly higher than values reported from Iran (0.01% and 0.46%) (31) and India (0.28% and 0.35%) (32), possibly reflecting differences in sample size and population characteristics.

Histopathologic correlation was available for 29 of 59 cases with significant cytologic abnormalities (Table 5). According to the Bethesda System (2001), LSIL cytologically corresponds to histologic mild dysplasia/CIN1, while HSIL corresponds to moderate to severe dysplasia (CIN2/CIN3) and carcinoma in situ (CIS) (Figure 2).

The diagnostic performance of Pap smear cytology for detecting epithelial abnormalities is summarized in Table 6. The test demonstrated the lowest sensitivity (60%), specificity (70%), and diagnostic accuracy (68.97%) for LSIL detection. A prior Iraqi study reported a Pap test sensitivity of 94%, specificity of 60%, and overall accuracy of 74% (33). In the present work, the overall sensitivity, specificity, positive predictive value (PPV), negative predictive value (NPV), and diagnostic accuracy were 76.19%, 80.30%, 55.17%, 91.38%, and 79.31%, respectively. Although sensitivity, specificity, and overall accuracy were moderately high and NPV was excellent, the relatively low PPV reflects the influence of the low prevalence of disease in the screened population. These results are comparable to those reported by Naik et al. (74.5%) (34), Saha et al. (79.1%) (35), Jain et al. (73.2%) (36), and Chaudhary et al. (76%) (37), whereas lower diagnostic accuracies were reported by Ashmita et al. (38) and Mallur et al. (39).

In developed nations, the introduction of HPV vaccination and organized screening programs has led to a 50%–75% reduction in cervical cancer–related mortality over the past five decades (40). In this study, the majority of intraepithelial lesions were observed in women aged 40–60 years, aligning with previous studies reporting increased risk of cervical abnormalities among women aged 45 years and older (41,42). Naik et al. (32) also observed a similar age distribution for intraepithelial lesions and malignancies. According to the World Health Organization, all women should undergo cervical cancer screening at least once before the age of 45 (43). The mean age of women screened in this study (36.97 years) highlights the need for increased awareness and earlier initiation of screening in this population.

This study has certain limitations, primarily its single-center design, which may limit generalizability to the entire Iraqi population. However, given that the laboratory serves patients from multiple regions of Iraq, this limitation is partially mitigated. Another limitation involves the use of both conventional and liquid-based cytology methods; however, no significant difference in diagnostic yield was observed between the two techniques.

Future studies should aim to assess the diagnostic performance, cost-effectiveness, and population impact of different cervical cancer screening methods and include HPV DNA testing and genotype identification. Such investigations would support the establishment of a national HPV vaccination and screening program to reduce cervical cancer incidence and mortality in Iraq.

## Conclusion

This study provides valuable insight into the prevalence and spectrum of cervical epithelial abnormalities among Iraqi women, revealing a 5.45% prevalence rate over a ten-year period, comparable to rates observed in other Middle Eastern populations. ASC-US was identified as the most common cytological abnormality, and lesions were most frequent among women aged 40–60 years. The Pap smear demonstrated moderate sensitivity, specificity, and overall diagnostic accuracy but a relatively low positive predictive value. The findings reinforce the importance of maintaining and expanding cervical cancer screening programs in Iraq, with emphasis on early detection, HPV genotyping, and preventive vaccination strategies.

## Recommendation

While the Pap smear remains a valuable screening tool, future studies should focus on adjunctive screening methods, such as HPV DNA testing and genotyping, to enhance detection rates and support the implementation of a nationwide HPV vaccination program. Further multi-center studies are recommended to provide a more comprehensive understanding of the epidemiology of cervical cancer in Iraq and to evaluate the impact of cultural and socioeconomic factors on screening uptake. Strengthening public health education and awareness programs regarding cervical cancer prevention and the importance of regular screening is also essential to reduce disease burden and mortality.

## List of abbreviations

AGC-NOS: Atypical glandular cells, not otherwise specified.

ASC-H: Atypical squamous cells – cannot exclude HSIL.

ASC-US: Atypical squamous cells of undetermined significance.

CIN: Cervical intraepithelial neoplasia. 

HPV: Human papilloma virus.

HSIL: High-grade squamous intraepithelial lesion.

LBC: Liquid-based cytology.

LSIL: Low-grade squamous intraepithelial lesion.

NPV: Negative predictive value. 

Pap smear: the Papanicolaou test.

PPV: Positive predictive value. 

SPSS: Statistical Package for Social Sciences.

WHO: The World Health Organization.

## Data Availability

The data supporting the results of this study are available upon request from the corresponding author.
